# IL-1R2 expression in human gastric cancer and its clinical significance

**DOI:** 10.1042/BSR20204425

**Published:** 2021-03-26

**Authors:** Maoling Yuan, Lei Wang, Hao Huang, Yuan Li, Xiao Zheng, Qi Shao, Jingting Jiang

**Affiliations:** 1 Department of Tumor Biological Treatment, The Third Affiliated Hospital of Soochow University, Changzhou, Jiangsu Province, China; 2 Cancer Immunotherapy Engineering Research Center of Jiangsu Province, Changzhou, Jiangsu Province, China; 3 Institute of Cell Therapy, Soochow University, Changzhou, Jiangsu Province, China; 4 Department of Geriatrics, The Third Affiliated Hospital of Soochow University, Changzhou, Jiangsu Province, China; 5 Department of Gastrointestinal Surgery, The Third Affiliated Hospital of Soochow University, Changzhou, Jiangsu Province, China

**Keywords:** Cancer progression, Gastric cancer, IL-1R2, Immunohistochemistry

## Abstract

**Background:** Interleukin-1 receptor type II (IL-1R2), also known as CD121b, is a member of the IL-1 receptor family. IL-1R2 acts as negative regulator of the IL-1 system, modulating IL-1 availability for the signaling receptor. IL-1R2 is abnormally expressed in many human inflammatory diseases and cancers, and has important clinical significance. The present study was designed to investigate IL-1R2 expression in human gastric cancer (GC) tissues and the associated clinical implications. **Methods:** Immunohistochemistry was used to identify the clinical significance and prognostic value of IL-1R2 expression in GC tissues. We investigated IL-1R2 expression in GC tissues, cells, and serum using real-time PCR (RT-PCR) and enzyme-linked immunosorbent assay (ELISA) assays. **Results:** IL-1R2 was highly expressed in GC tissues, and the overall survival in patients with advanced GC and high IL-1R2 expression was significantly poorer than that in patients with advanced GC and low IL-1R2 expression. Moreover, *IL-1R2* mRNA levels in GC tissues and most GC cells were higher than those in para-cancer tissues and GES1 human gastric mucosal epithelial cells. The level of plasma-soluble IL-1R2 in GC patients was higher than that of the healthy control group. **Conclusion:** Increased IL-1R2 levels are involved in the initiation and progression of human GC, and IL-1R2 might be employed to develop immunotherapeutic approaches targeting GC.

## Introduction

Gastric cancer (GC) is the fifth most commonly diagnosed cancer and the third leading cause of cancer-associated mortality in men and women worldwide [1]. Patients with GC exhibit high metastasis and mortality rates and low early diagnosis rates [2]. Most patients with GC have advanced-stage disease at diagnosis and have missed the optimum surgical window. Although there are many treatment methods for GC, including surgery, chemotherapy, radiotherapy, and immunotherapy, the 5-year survival rate of patients remains poor [3]. Therefore, it is important to identify new targets for the diagnosis, treatment, and prognosis of GC.

The interleukin-1 receptor type II (*IL-1R2*) gene is located on the long arm of chromosome 2 at band 2q12 in humans. The IL-1R2 protein is the predominant IL-1 receptor, and is 398 amino acids in length [4]. However, IL-1R2 differs from all other members of the IL-1 receptor family because it lacks a TIR domain and only has a short cytoplasmic tail of only 29 amino acids in length. As a decoy receptor, IL-1R2 cannot signal by competitive binding to IL-1β and preventing its binding to IL-1R1 [5,6]. IL-1R2 is mainly expressed in neutrophils, B cells, monocytes, and macrophages. Plasma levels of soluble IL-1R2 are between 5 and 10 ng/ml in healthy donors and increase in patients with infectious conditions [7]. IL-1R2 plays a role in a variety of diseases, including chronic skin inflammation, arthritis, endometriosis, and heart transplantation or autoimmune myocarditis [7]. Moreover, IL-1R2 overexpression is observed in a variety of tumors, and is indicative of poor prognosis, in breast cancer [8], colorectal cancer [9], pancreatic cancer [10], lung cancer [11], and oral cancer [12].

Here, we investigated the clinical significance of IL-1R2 expression in human GC tissues and plasma, the relationship between IL-1R2 expression and clinicopathological factors, and evaluated its prognostic value.

## Materials and methods

### Patients and sample collection

The GC tissue array (Catalog number: HStmA180Su15, 98 cancer tissues, 82 normal tissues) was purchased from Shanghai Outdo Biotech Co., Ltd. (Shanghai, China). The array contained samples from 98 GC patients that underwent surgery between July 2006 and April 2007. The age of the patients ranged from 41 to 81 years, with a median age of 65 years. Patients were followed up for 8.2–9.0 years. No patient received preoperative radiotherapy or chemotherapy. All patient surgical specimens were confirmed as GC by pathologists using Hematoxylin and Eosin (H&E) staining. After excluding the incomplete tissue points and several missing tissue points when performing heated antigen retrieval, 88 cancer cases and 75 normal tissues were analyzed. The correlation between GC tissue IL-1R2 expression levels and the patients’ clinical parameters are listed in Table 1.

**Table 1:** Relationship of IL-1R2 expression level in GC tissues and clinicopathological parameters

Clinical parameters	Cases	IL-1R2 expression level		χ^2^	*P*-value
		*H-score ≤* 80	*H-score >* 80		
Gender					
Male	54	29	25	0.006	0.939
Female	33	18	15		
Age (years)					
≤65	41	23	18	0.134	0.714
>65	46	24	22		
Tumor size (cm)					
≤5.5	45	23	22	0.677	0.411
>5.5	40	24	16		
Pathological stage					
I + II	15	7	8	0.395	0.530
III + IV	72	40	32		
T stage					
T1+T2	15	6	9	1.435	0.231
T3+T4	72	41	31		
Lymph node metastasis					
without	23	13	10	0.044	0.833
with	63	34	29		
TNM stage					
I + II	37	17	20	1.986	0.159
III + IV	49	30	19		

### Antibodies and major reagents

The goat polyclonal antibody against human IL-1R2 (AF263, diluted in 1:1) was purchased from R&D Systems (Minneapolis, MN, U.S.A.). HRP-conjugated anti-goat IgG polymer (PV-9003) was obtained from ZSGB-BIO (Beijing, China). The Human IL-1R2 Quantikine ELISA Kit (DR1B00) was purchased from R&D Systems (Minneapolis, MN, U.S.A.). The RNeasy Mini Kit was supplied by Qiagen (Valencia, CA, U.S.A.), and SYBR Green Master Mix kits were provided by TaKaRa (Dalian, China). DMEM and fetal bovine serum (FBS) were purchased from Gibco (Cambrex, MD, U.S.A.).

### Immunohistochemistry

Immunohistochemistry assays were used to detect IL-1R2 protein expression levels in human GC and normal gastric tissues. The paraffin-embedded tumor tissue microarray block was sectioned into 3 μm sections, dewaxed with xylene, and rehydrated with a series of graded alcohols. Tissue sections were heated at 100°C for 30 min in EDTA (1 mM, pH 9.0) for antigen retrieval. After cooling, the tissue sections were immersed in 0.3% hydrogen peroxide solution for 15 min to block endogenous peroxidase activity, washed with PBS for 5 min, and blocked with 3% BSA solution at room temperature for 20 min. The goat anti human IL-1R2 polyclonal antibody was incubated overnight at 4°C, and then incubated with the HRP-conjugated anti-goat IgG polymer. Diaminobenzene was used as the chromogen and Hematoxylin was used as the nuclear counterstain. Finally, the sections were dehydrated, cleared, and mounted.

### Evaluation of immunohistochemical staining

Two pathologists with no knowledge of the patients’ information independently examined the stained sections. IL-1R2 staining in the GC tissue array was assessed according to the H-score method as described in our previous report [13]. The results were calculated as: H-score = %tumor cells unstained × 0 + %tumor cells stained weak × 1 + %tumor cells stained moderate × 2 + %tumor cells stained strong × 3. H-scores ranged from 0 (100% negative tumor cells) to 300 (100% strongly stained tumor cells). Results from the two pathologists were averaged and used for statistical analysis.

### Enzyme-linked immunosorbent assay

The enzyme-linked immunosorbent assay (ELISA) was performed to detect IL-1R2 levels in GC patients and healthy people. Twenty-eight serum samples from healthy people and 50 serum samples from patients with GC were obtained from the Third Affiliated Hospital of Soochow University. Informed consent was obtained from all patients, and the study was approved by the institution’s ethics committee. All assays were performed according to the manufacturer’s instructions.

### Cell culture

GES1 and human GC cell lines (AGS, MGC803, BGC823, and SGC7901) were obtained from the Chinese Academy of Sciences, Shanghai Institutes for Biological Sciences, and were cultured in standard DMEM supplemented with 10% FBS and antibiotics (100 U/ml of penicillin and 100 μg/ml of streptomycin) under standard culture conditions (5% CO_2_, 37°C).

### Real-time PCR

Real-time PCR (RT-PCR) was performed to evaluate *IL-1R2* mRNA expression in GC tissues and cells. GC tissue and para-cancer tissue was obtained from the Gastrointestinal Surgery Department of the Third Affiliated Hospital of Soochow University. Informed consent was obtained from all patients, and the study was approved by the institution’s ethics committee. Total RNA was extracted from GC tissues and cells using TRIzol reagent (Invitrogen) and reverse transcribed into cDNA using an RT reaction kit (Promega). RT-PCR was performed to detect relative RNA expression using the SYBR green method and an ABI 7500 real-time PCR system (Applied Biosystems, U.S.A.). Human GAPDH was selected as an internal reference gene. GAPDH and IL-1R2 primer sequences used were: GAPDH forward primer, 5′-GGAGCGAGATCCCTCCAAAAT-3′; GAPDH reverse primer, 5′-GGCTGTTGTCATACTTCTCATGG-3′; IL-1R2 forward primer, 5′-GCCAATGACACCCACATAGAGAGC-3′, and IL-1R2 reverse primer, 5′-GGAAGAGCGAAACCCACAGAGTTC-3′. The relative IL-1R2 mRNA expression was calculated using the 2^−ΔΔ*C*_T_^ method.

### Statistical analysis

All data were analyzed using the GraphPad Prism 8.0 software package (GraphPad Software, Inc., San Diego, U.S.A.) and R 3.6.3. IL-1R2 protein expression in GC and normal tissue, IL-1R2 mRNA expression in GC and para-cancer tissue, and serum IL-1R2 levels in GC and healthy controls were tested using the unpaired Student’s *t* test. The association between IL-1R2 expression and the clinical pathological characteristics was assessed using the chi-square test. R package survival was used for single-factor and multi-factor analyses. Kaplan–Meier and log-rank tests were used to compare the overall survival rate of patients with different clinicopathological parameters. Cox proportional hazards model was used to estimate the risk (HR) among different clinicopathological parameters, IL-1R2 expression and death risk with 95% confidence interval (CI). A *P*-value <0.05 was considered statistically significant.

## Results

### IL-1R2 expression of in human GC tissues and normal gastric tissues

**Figure 1:**
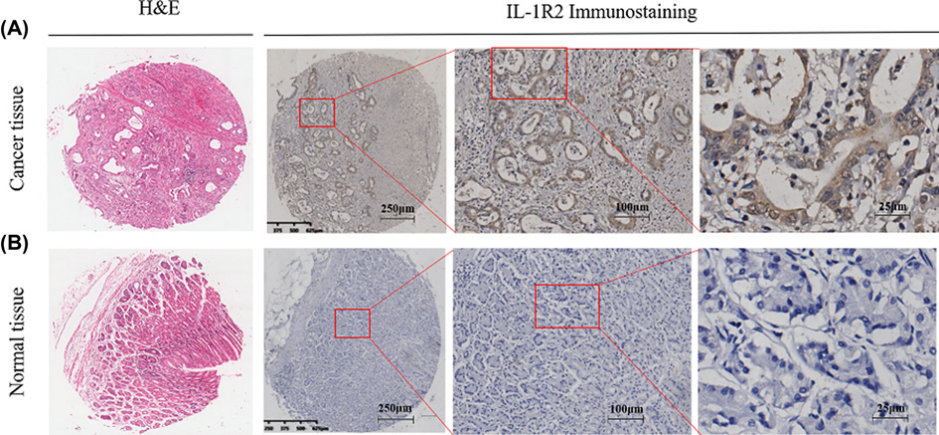
Immunohistochemical staining of IL-1R2 in GC tissues and normal gastric tissue (**A**) High IL-1R2 expression in human GC tissues, and positive staining of IL-1R2 might be found in the GC cell cytoplasm. (**B**) The IL-1R2 was not expressed in normal gastric tissues.

**Figure 2:**
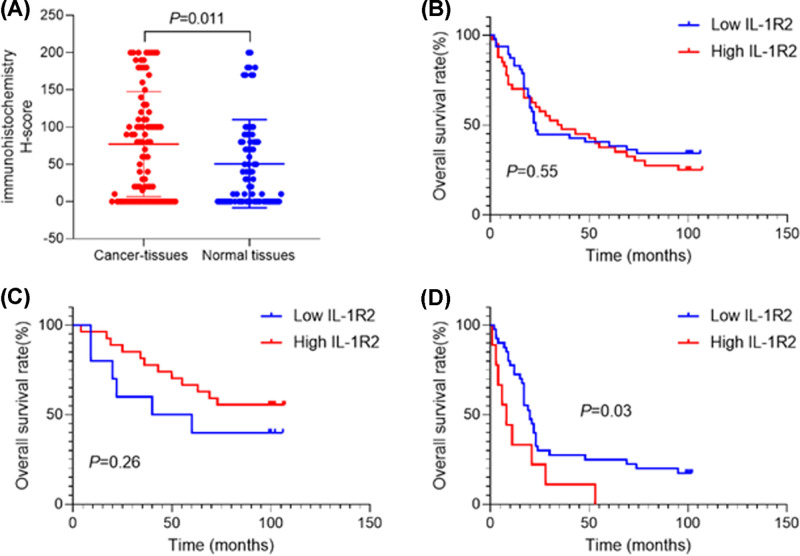
The relationship between the overall survival of GC patients and IL-1R2 expression (**A**) H-score of IL-1R2 staining in GC tissues was significantly higher than that in normal gastric tissues. (**B**) IL-1R2 expression was not associated with overall survival in GC patients. (**C**) IL-1R2 expression was not associated with overall survival in GC patients of TNM stages I and II. (**D**) Overall survival in TNM stages III and IV patients with high IL-1R2 expression was significantly poorer than that in patients with low IL-1R2 expression (*P*=0.03).

Immunohistochemical staining was used to detect IL-1R2 expression in GC tissues and normal gastric tissues. IL-1R2 expression was higher in GC tissues than in normal gastric tissue, and IL-1R2 positive staining was mainly located on the GC cell cytoplasm (Figure 1A). No, or weak, staining was observed in normal gastric tissues (Figure 1B). The H-score of IL-1R2 staining in GC tissues was significantly higher than that in normal tissues (*P*=0.011, Figure 2A). However, there was no correlation between IL-1R2 expression and any clinical parameter in GC patients (Table 1).

### Prognostic value of IL-1R2 expression in human GC

To study the relationship between IL-1R2 expression and prognosis in GC, we performed Kaplan–Meier survival analysis. The results showed that IL-1R2 expression was not associated with overall survival in GC patients (*P*=0.55, Figure 2A). Overall survival in TNM stages III and IV patients with high IL-1R2 expression was significantly poorer than that in patients with low IL-1R2 expression (*P*=0.03, Figure 2D). Multi-factor Cox analysis indicated that increased age (HR = 1.0324, 95% CI: 1.0031–1.062, *P*=0.030), TNM stage III + IV (HR = 4.489, 95% CI: 1.8343–10.986, *P*=0.001), and high IL-1R2 expression (HR = 2.151, 95% CI: 1.198–3.861, *P*=0.010) were independent prognostic factors of GC (Table 2).

**Table 2:** Univariate and multivariate analyses of clinicopathological characteristics for overall survival

Characteristics	Univariate analysis		Multivariate analysis	
	HR (95% CI)	*P*-value	HR (95% CI)	*P*-value
Gender				
Male/female	1.224 (0.734–2.04)	0.438	1.210 (0.687–2.131)	0.510
Age (years)				
>65/≤65	1.018 (0.990–1.047)	0.217	1.032 (1.003–1.062)	**0.030**
Tumor size (cm)				
>5.5/≤5.5	1.810 (1.088–3.011)	**0.022**	1.690 (0.942–3.029)	0.078
Pathological stage				
III + IV/I + II	2.720 (1.168–6.333)	**0.020**	1.958 (0.792–4.840)	0.146
T stage				
T3+T4/T1+T2	2.680 (1.150–6.243)	**0.022**	1.908 (0.793–4.595)	0.149
Lymph node metastasis				
with/without	2.548 (1.320–4.918)	**0.005**	0.991 (0.377–2.606)	0.985
TNM stage				
III + IV/I + II	3.308 (1.888–5.795)	**0.000**	4.489 (1.834–10.986)	**0.001**
IL1R2 expression				
High/Low	1.159 (0.701–1.917)	0.566	2.151 (1.198–3.861)	**0.010**

Bold signifies *P*<0.05.

### Serum IL-1R2 levels in of GC patients

**Figure 3:**
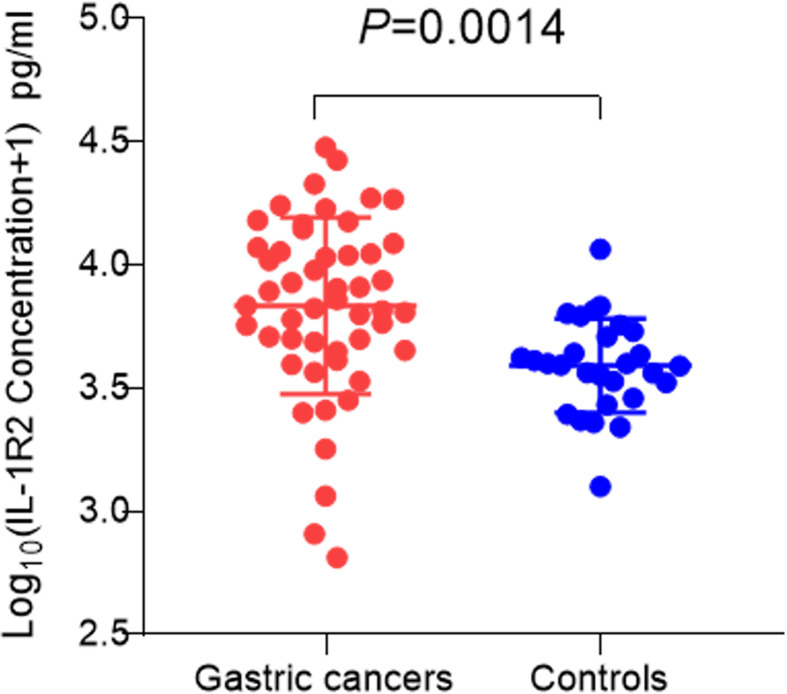
The level of IL-1R2 protein in serum of GC patients and controls ELISA analysis indicated that the serum level of IL-1R2 in GC patients was significantly higher than that in healthy controls.

To detect the serum IL-1R2 levels of GC patients, we analyzed the levels of serum soluble IL-1R2 in GC patients (*n*=50) and healthy controls (*n*=28). Using ELISA, we found that the serum IL-1R2 levels in GC patients were significantly higher than that in healthy controls (*P*<0.05, Figure 3).

### IL-1R2 mRNA levels in GC tissues and GC cells

**Figure 4:**
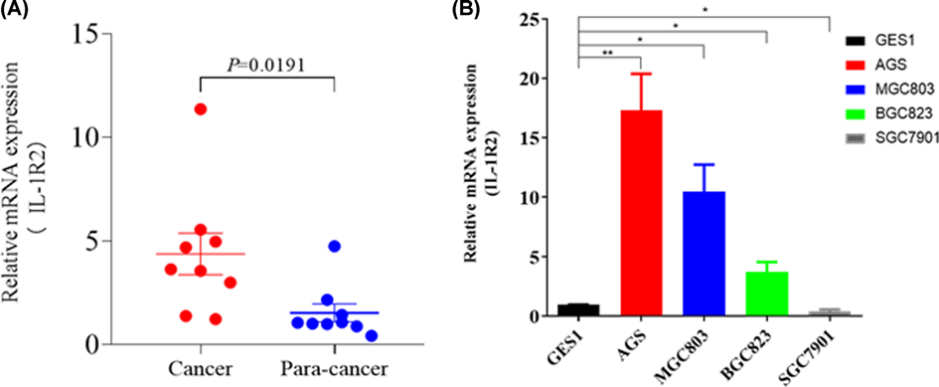
The levels of IL-1R2 mRNA in GC tissues and GC cells (**A**) *IL-1R2* mRNA levels were higher in GC tissues than in para-cancer tissues (*P*=0.0191). (**B**) The IL-1R2 mRNA levels in AGC, MGC803, and BGC823 cells was higher than that in GES1 (*P*<0.01, *P*<0.05 and *P*<0.05, respectively), however, the IL-1R2 mRNA level in SGC7901 cell was lower than that in GES1 (*P<0.05*). *P<0.05, **P<0.01.

*IL-1R2* mRNA expression in the GC and para-cancer tissues of nine patients with GC was measured by RT-PCR. *IL-1R2* mRNA levels were higher in GC tissues than in para-cancer tissues (*P*=0.0191, Figure 4A). We detected *IL-1R2* mRNA levels in GC cells. RT-PCR revealed that the IL-1R2 mRNA levels in most GC cells (AGC, MGC803, and BGC823; *P*<0.01, *P*<0.05, and *P*<0.05, respectively) were higher than that in GES1, with the exception of SGC7901 (*P*<0.05).

## Discussion

IL-1 is the key mediator of innate and adaptive immunity. IL-1 plays a key role in promoting inflammatory response by shaping different components of the tumor microenvironment, including tumor infiltration, myeloid cell recruitment, angiogenesis, and inhibition of anti-tumor immunity [14]. IL-1 plays a major role in the development of several autoimmune diseases and cancers, including type 2 diabetes [15], Alzheimer’s disease [16], esophageal cancer [17], and breast cancer [8]. Therefore, inhibition of IL-1 signal transduction is an important therapeutic approach for both cancer and autoimmune diseases [18], and IL-1R2 can inhibit IL-1 signal transduction. In the IL-1 negative regulatory system, IL-1R2 acts as a decoy receptor and a negative regulator, suggesting that it plays a crucial role in the IL-1-mediated immune response [19]. IL-1R2 exists in two isoforms, membrane-bound protein and soluble protein [20]. Plasma soluble IL-1R2 levels in healthy blood donors are between 5 and 10 ng/ml, and plasma soluble IL-1R2 levels are elevated in infected patients [21].

IL-1R2 is expressed in a variety of diseases and tumors, and plays a role in promoting cancer in most tumors. The expression of intracellular IL-1R2 in human colorectal cancer cells is higher than that in normal colon cells. Intracellular IL-1R2 regulates IL-6 and VEGF-A expression and the migration and proliferation of colorectal cancer cells [9]. Zhang et al. showed that IL-1R2 is up-regulated in breast cancer tissues, and IL-1R2 increases BMI1 deubiquitination and stability by binding to and enhancing the activity of ubiquitin-specific protease 15, promoting breast cancer cell proliferation and invasion. Meanwhile, IL-1R2‐neutralizing antibody significantly inhibits cancer cell growth, invasion, and chemoresistance *in vitro* [8]. Moreover, the level of IL-1R2 in infiltrating Treg cells is higher in colorectal cancer tissues than in normal tissues. IL-1R2 up-regulation may be another mechanism through which tumor-resident Treg cells inhibit anti-tumor immune response through neutralizing effector cell IL-1β function [22]. These studies suggest that IL-1R2 plays an important role in regulating the biological behavior of cancer cells. Therefore, IL-1R2 is a potential clinical biomarker for human cancer, and a potential new therapeutic target for the treatment of cancer.

In the present study, IL-1R2 expression in GC and normal gastric tissues was detected using tissue microarray and immunohistochemistry. Our results show that IL-1R2 expression in GC tissues is higher than that in normal gastric tissues, and that there was no significant correlation between IL-1R2 expression and the clinicopathological characteristics of GC. Survival analysis revealed that IL-1R2 expression was not associated with overall survival in patients with GC. However, in patients with TNM stages III and IV GC, overall survival was significantly poorer in those with high IL-1R2. We investigated the expression of IL-1R2 in GC tissues, cells, and serum using RT-PCR and ELISA assays. We found that IL-1R2 mRNA levels was higher in GC tissues than in para-cancer tissues, and that the level of IL-1R2 mRNA in most GC cells was higher than that in GES1 control cells. The plasma soluble IL-1R2 levels were higher in the GC group than in the healthy control group. Collectively, our results show that increased IL-1R2 is involved in the progression of human GC. The potential contribution of IL-1R2 examination in immunotherapeutic approaches against human GC and the underlying mechanisms of IL-1R2 in GC progression need to be further investigated.

## Conclusions

Our results suggest that increased IL-1R2 is involved in the initiation and progression of human GC, suggesting that IL-1R2 could be a potential predictor of therapy against human GC.

## Data Availability

All the data presented in the present study are available from the corresponding author upon reasonable request.

## Abbreviations

CI: confidence interval
ELISA: enzyme-linked immunosorbent assay
GC: gastric cancer
HR: Hazard Ratio
HRP: Horseradish Peroxidase
IL-1R2: interleukin-1 receptor type II
RT-PCR: real-time PCR
TIR: Toll/Interleukin-1 receptor
VEGF-A: Vascular endothelial growth factor-A
